# A novel uranyl ion-binding peptide enhances U(VI) adsorption capacity and selectivity of *Saccharomyces cerevisiae*

**DOI:** 10.3389/fmicb.2026.1869418

**Published:** 2026-07-13

**Authors:** Bo Liang, Jiaqing Luo, Baifeng Zhou, Peiyuan Xu, Yufei Feng, Hongyan Zhang, Min Li

**Affiliations:** 1 Analysis and Test Center, Lingnan Normal University, Zhanjiang, China; 2 Life Science and Technology School, Lingnan Normal University, Zhanjiang, China

**Keywords:** biosorption, selectivity, U(VI), uranyl ion-binding peptide, yeast surface display

## Abstract

The lack of effective and selective adsorbents for uranium (U(VI)) removal has become a growing concern in the nuclear industry. In this study, a novel uranyl-binding peptide was isolated from a phage-displayed random heptapeptide library through three rounds of biopanning. Sequence analysis revealed enrichment of basic and polar amino acids, including arginine and serine. One of the identified peptides, designated 7–5, was cloned into the yeast surface display vector pYD1 and expressed in *Saccharomyces cerevisiae* EBY100, generating the recombinant strain EBY100-7-5. Under optimal conditions (pH 4.73), the engineered strain exhibited a maximum U(VI) adsorption capacity of 79.33 ± 3.39 mg/g, representing a 22.69% increase compared to the control strain EBY100. The adsorption kinetics followed a pseudo-second-order model, and the equilibrium data were well described by the Freundlich isotherm. Thermodynamic analysis indicated that the adsorption process was spontaneous and endothermic. In the presence of 11 competing metal ions, EBY100-7-5 showed selective U(VI) adsorption, with significantly higher uptake for U(VI) than for other metal ions. Scanning electron microscopy combined with energy-dispersive X-ray spectroscopy (SEM-EDS) revealed particulate deposits and characteristic U(VI) signals on the cell surface after adsorption, confirming that the engineered yeast cells served as the primary active sites. Collectively, these results demonstrate that the uranyl-binding peptide confers high adsorption capacity, rapid kinetics, and strong selectivity to EBY100-7-5, presenting this engineered strain as a promising biosorbent for the efficient treatment of uranium-containing radioactive wastewater.

## 1 Introduction

The rapid expansion of the nuclear industry has driven growing uranium demand and the concomitant generation of large volumes of uranium-containing radioactive wastewater from mining, fuel processing, and related operations (Liu et al., 2025). Direct discharge of untreated wastewater poses serious threats to ecological safety and human health (Kaur Guron et al., 2025; Ashish et al., 2024). Moreover, as a finite strategic resource, uranium recovery and reuse are essential for the sustainable development of the nuclear industry. Consequently, developing efficient, cost-effective, and environmentally friendly technologies for uranium removal and recovery has become a research priority (Jun et al., 2021).

Conventional treatment methods, including ion exchange and chemical precipitation, face limitations such as high operating costs and secondary pollution (Gandhi et al., 2022; Chandrakar et al., 2025). Biosorption technology has emerged as a promising alternative due to its low cost, high efficiency, and environmental compatibility (Aslam et al., 2025; Guillén et al., 2026). Among various biosorbents, yeast is particularly attractive because it is safe, easy to cultivate, and grows rapidly (Dhanya et al., 2025).

However, natural microbial materials exhibit limited adsorption capacity, hindering efficient uranyl ion enrichment. To overcome this limitation, genetic engineering has been employed to express metal-binding proteins or peptides in microbial cells such as *Saccharomyces cerevisiae* (Li L. et al., 2025; Jiang et al., 2024). Intracellular expression of binding peptides like metallothionein improves adsorption efficiency for certain heavy metals, including Cd^2+^ and Pb^2+^ (Akkurt et al., 2024; Oliveira et al., 2020). However, this approach depends on cell viability, and heterologous peptides are often unstable and prone to intracellular degradation, limiting its practical applicability.

Cell surface display technology offers a solution to this problem. By fusing a metal-binding peptide-encoding gene with an anchor protein gene, the target peptide is displayed on the microbial cell surface. Surface-displayed binding peptides can directly interact with metal ions in the environment, thereby increasing adsorption rates and capacities while facilitating metal recovery (Granuzzo et al., 2025; Wang et al., 2025). This technology has been successfully applied to adsorb Cd^2+^, Hg^2+^, and Cr^6+^ (Lu et al., 2023; Hu et al., 2024; Yang et al., 2024), but reports on uranyl ions remain limited.

Furthermore, uranium-containing wastewater typically contains multiple competing metal ions, and the poor selectivity of available adsorbents severely limits their uranium adsorption performance (Lin et al., 2026; Meziane et al., 2026). Addressing this issue requires binding peptides that specifically recognize target metal ions. Naturally occurring metal-binding peptides such as metallothionein lack diversity, and their selectivity toward uranyl ions is unknown, making one-by-one screening inefficient.

In contrast, phage-displayed random peptide library technology provides a powerful high-throughput tool for screening specific targeting peptides (Chen et al., 2025; Xu et al., 2021; Dantas et al., 2025). This approach has successfully identified selective binding peptides for Ni^2+^, Cd^2+^, Co^2+^, Cu^2+^, and Pb^2+^ (Sieber et al., 2025; Techert et al., 2025; Maass et al., 2024; Sosnowska et al., 2025; Wang et al., 2024; Korkmaz and Kim, 2024), but no studies have yet reported the screening and identification of uranyl ion-binding peptides.

In this study, a phage-displayed heptapeptide library was subjected to biopanning against uranyl ions to enrich and isolate specific uranyl-binding peptides. The selectivity of the obtained peptides was evaluated in the presence of competing metal ions. The genes encoding the screened peptides were then expressed on the surface of *S. cerevisiae*, and the adsorption performance of the engineered yeast cells toward uranyl ions was characterized. This study provides a novel candidate biosorbent for the treatment of uranium-containing wastewater and the recovery of uranium resources.

## 2 Materials and methods

### 2.1 Culture media

All culture media were prepared according to the manufacturer’s instructions for the Ph. D.™ -7 Phage Display Peptide Library Kit. Concentrations are given in g/L: LB liquid medium (10 peptone, 5 yeast extract, 5 NaCl, pH 7.0), with 15 agar for solid medium; YPD medium (10 yeast extract, 20 peptone, 20 glucose), with 15 agar for solid medium; Top agar (10 peptone, 5 yeast extract, 5 NaCl, 7 agar); YNB-CAA medium (6.7 YNB with ammonium sulfate, 5 acid-hydrolyzed casein), supplemented with 20 glucose for cultivation or 20 galactose for induction; and MD selective medium (6.7 YNB, 20 glucose, 0.1 leucine, 15 agar) for screening transformants.

### 2.2 Strains and reagents

*S. cerevisiae* EBY100 and plasmid pYD1 were kindly provided by the Institute of Genetics and Developmental Biology, Chinese Academy of Sciences (Boder and Wittrup, 1997). *Escherichia coli* JM109 was maintained in our laboratory (Yanisch-Perron et al., 1985). The Ph. D.- 7TM phage display library kit was purchased from New England Biolabs. Plasmid Mini Kit I and Yeast Plasmid Kit were purchased from Omega. *Ex Taq* DNA Polymerase was purchased from Takara Biotechnology Co., Ltd. Chelating Sepharose Fast Flow resin was purchased from Pharmacia Biotech. Primers, heptapeptide synthesis, and DNA sequencing services were provided by Suzhou Jinweizhi Biotechnology Co., Ltd. Restriction endonucleases, calf thymus ssDNA, Polyethylene glycol (PEG) 3350, Xgal, IPTG, and other reagents were purchased from Sangon Biotech Co., Ltd. Mouse anti-His primary antibody and Alexa Fluor 488-conjugated secondary antibody were purchased from Saigao Wei Biotechnology Co., Ltd. (China). All other reagents were of domestic analytical grade.

### 2.3 Preparation of uranyl ion-chelated resin

A 100 μL volume of resin was placed in a 1.5 mL centrifuge tube and centrifuged at 15,000 × g for 5 min, and the supernatant was discarded. After four washes with sterile water, the resin was added to 150 μL of 20 μg/mL uranium standard solution and agitated overnight at 150 rpm. The mixture was then centrifuged at 15,000 × g for 10 min, and the supernatant was removed. The obtained uranyl ion-chelated resin (M^U+^) had a uranium loading of approximately 5.3 μg/mL resin. Resin treated with sterile ultrapure water served as the control (M^−^).

### 2.4 Screening of uranyl ion-binding peptides

#### 2.4.1 Biopanning of the phage library

M^U+^ and M^−^ resins (100 μL each) were washed twice with 1,000 μL of 0.1% TBST (pH 7.4). Then, 10 μL of the phage peptide library (2.0 × 10^13^ pfu/mL) was added, and the mixtures were shaken at 25 °C and 100 rpm for 1 h, then centrifuged at 360 × g and 4 °C for 30 s. After centrifugation, 1,000 μL of 0.1% TBST was added, and the mixtures were shaken at 100 rpm for 1 min and centrifuged. This wash procedure was repeated twice. Next, 500 μL of 20 mM imidazole (ID) was added, followed by centrifugation. This step was performed twice. The samples were then washed five times with 1,000 μL of 0.1% TBST. Finally, 300 μL of 200 mM ID was added, and the bound phages were eluted by shaking for 20 min. This elution was repeated, and the two eluates were combined for titer determination (Wang et al., 2024).

The eluates were amplified in *E. coli* ER2738, and the next screening round was performed. In rounds 2 and 3, the washing conditions were modified as follows: four washes with 1,000 μL 0.3% TBST, four washes with 500 μL of 20 mM ID, and finally eight washes with 1,000 μL of 0.3% TBST. Elution was carried out as in round 1. A total of three rounds of biopanning were conducted. Titers were recorded after each round, and the P/N ratio was calculated as the ratio of the eluted phage titer from U(VI)-chelated resin (M^U+^, positive) to that from the control resin (M^−^, negative).

#### 2.4.2 Determination of peptide affinity toward uranyl ions

The affinity determination was performed according to the manufacturer’s instructions for the Ph. D.-7TM phage display library. After the third biopanning round, phages were amplified in *E. coli* ER2738, and 20 independent blue plaques were randomly selected from plates with fewer than 100 plaques. Each plaque was inoculated into an overnight *E. coli* ER2738 culture for amplification. A 10 μL aliquot of each amplified culture was mixed with M^U+^ and M^−^ resins, eluted with ID, and the phage titer was determined to calculate the P/N ratio.

#### 2.4.3 Extraction and sequencing of phage DNA

PEG 3350 was added to the phage amplification solution for overnight precipitation at 4 °C. The precipitate was resuspended in TE buffer, then treated with proteinase K (50 μg/mL) and SDS (0.5% w/v) to lyse the capsid. DNA was extracted with phenol/chloroform, precipitated with sodium acetate and ethanol, washed, air-dried, dissolved in TE buffer, and submitted for sequencing (Jakočiūnė and Moodley, 2018).

#### 2.4.4 Selectivity analysis of the binding peptides toward uranyl ions

A mixed metal ion solution was prepared using SmCl₃·6H₂O, Sr.(NO₃)₂, CsCl, CeCl₃, and Gd₂O₃, each at a concentration of 20 μg/mL. Mixed metal ion-loaded chelating resin (M^H+^) was prepared as described in Section 2.3. M^H+^, M^U+^, and M^−^ resins (100 μL each) were washed twice with 0.1% TBST, mixed with the phage amplification solution, and incubated at 25 °C and 130 rpm for 1 h. The samples were then washed sequentially: 10 times with 0.1% TBST, 8 times with 20 mM ID, and 10 times with 0.1% TBST. Bound peptides were eluted twice with 300 μL of 200 mM ID, and the eluates were combined. The phage titer of the combined eluates was determined, and the P/N ratios for M^U+^/M^−^ and M^H+^/M^−^ were calculated.

### 2.5 Surface display of uranyl ion-binding peptides on yeast cells

#### 2.5.1 Construction of the surface display vector

The heptapeptide sequence of clone 7-5 (LSQRMQR) was used to synthesize the complementary DNA oligonucleotide 5′-TTG TCT CAA AGA ATG CAA AGA-3′. *Bam*HI and *Xho*I restriction sites were added to the 5′ and 3′ ends, respectively, to facilitate directional cloning. The resulting fragment was inserted in-frame into the pYD1 vector downstream of the *Aga2* gene. The expected molecular weight of the fusion protein is approximately 19 kDa.

#### 2.5.2 Transformation and identification of recombinant yeast cells

The recombinant plasmid was introduced into competent *S. cerevisiae* EBY100 cells using the lithium acetate whole-cell transformation method (Gietz and Schiestl, 1991). After transformation, the cell suspension was spread on MD plates and incubated at 30 °C for 48 h to obtain single colonies. A single clone was picked and inoculated into YNB-CAA liquid medium containing 2% glucose, then shaken overnight at 30 °C and 170 rpm. The cells were harvested, and plasmid DNA was extracted using a yeast plasmid extraction kit. The extracted DNA served as the template for PCR identification.

The PCR reaction mixture (25 μL) contained 1 μL of each primer (10 μmol/L): PF (5′-AGTAACGTTTGTCAGTAATTGC-3′) and PR (5′-GTCGATTTTGTTACATCTACAC-3′), 0.1 μL template DNA, 2 μL of 2.5 mmol/L dNTPs, 2.5 μL of 10 × *Ex Taq* Buffer, and 0.25 μL of *Ex Taq* polymerase, with ddH₂O added to volume. The amplification program was as follows: pre-denaturation at 94 °C for 3 min; 30 cycles of denaturation at 94 °C for 30 s, annealing at 54 °C for 30 s, and extension at 72 °C for 45 s; followed by a final extension at 72 °C for 5 min. The amplified products were verified by sequencing, and positive transformants with the correct sequence were selected.

#### 2.5.3 Induced expression of the engineered strain and flow cytometric analysis

Induced expression of the engineered strain was performed according to the pYD1 manual (Invitrogen). Cells were harvested at 36 h post-induction, washed twice with PBS, and incubated with mouse anti-His primary antibody (1:1000) for 30 min on ice. After washing, cells were incubated with Alexa Fluor 488-conjugated goat anti-mouse IgG secondary antibody (1:500) for 30 min on ice in the dark. Non-induced cells (0 h) served as a negative control. After two additional washes, cells were resuspended in 40 μL PBS and analyzed using a flow cytometer.

### 2.6 Adsorption experiments

The engineered yeast strain EBY100-7-5, along with control strains EBY100 and EBY100-pYD (carrying empty vector pYD1), were induced for 36 h and then freeze-dried. For each adsorption experiment, 30 mg of freeze-dried cells were added to 50 mL of U(VI) solution at a specified initial concentration in 150 mL Erlenmeyer flasks. The mixtures were shaken at 150 rpm and 298.15 K (unless otherwise specified for thermodynamic experiments). After adsorption, the mixtures were centrifuged at 8,000 × g for 10 min to separate the biomass. The residual U(VI) concentration in the supernatant was determined using the Arsenazo III method (Jauberty et al., 2013), and the adsorption capacity *q*_e_ (mg·g^−1^) was calculated according to Equation 1:

$$q_{e}=(C_{0}-C_{e})V/W$$ (1)

where *C_0_* is the initial U(VI) concentration (mg/L), *C_e_* is the U(VI) concentration at adsorption equilibrium (mg/L), *V* is the solution volume (L), and *W* is the adsorbent mass (g).

Kinetic adsorption experiments were conducted at an initial U(VI) concentration of 50 mg/L, pH 4.73, and 298.15 K. Samples were taken at various time intervals (0.25, 0.75, 1, 2, 3, 4, 5, 6 h). Isothermal adsorption experiments were carried out at 298.15 K and pH 4.73, with initial U(VI) concentrations set at 10, 20, 30, 40, 50, 60, 70, 80, 90, and 100 mg/L. The mixtures were shaken for 3 h before centrifugation and analysis. Thermodynamic experiments were performed at four different temperatures, specifically 288.15 K, 298.15 K, 308.15 K, and 313.15 K, under otherwise identical conditions including an initial U(VI) concentration of 50 mg/L, pH 4.73, and a contact time of 3 h. The model fitting and parameter calculation methods are detailed in Li M. et al. (2025), Li L. et al. (2025).

To evaluate selective U(VI) adsorption, a simulated wastewater containing 11 metal ions (Sm(III), Gd(III), Ce(III), La(III), Cs(I), Co(II), Sr.(II), Ni(II), Zn(II), Mn(II), and U(VI)) was prepared, each at an initial concentration of 50 mg/L. The pH was adjusted to 4.73. All metal ions were tested simultaneously in a single mixed competitive system. After adsorption, the concentration of each metal ion in the supernatant was simultaneously determined using inductively coupled plasma mass spectrometry (ICP-MS).

### 2.7 Scanning electron microscopy and energy dispersive spectroscopy analysis

The surface morphology of the engineered yeast cells before and after U(VI) adsorption was observed using scanning electron microscopy (SEM; Nova NanoSEM 450, FEI Company, United States). Elemental composition analysis of specific regions was performed using energy dispersive spectroscopy (EDS; X-Max80, Oxford Instruments, UK).

### 2.8 Data processing

All experiments were performed in three biologically independent replicates (*n* = 3). Data are shown as mean ± SD. Raw data processing and preliminary analysis were performed using Microsoft Excel 2013, and data fitting was conducted using Origin 2025. Statistical analyses were performed using SPSS. Two-tailed independent samples *t*-test was used for two-group comparisons, and one-way ANOVA with Tukey’s HSD *post hoc* test was used for three or more groups. Statistical significance was set at *p* < 0.05.

## 3 Results

### 3.1 Screening of uranyl ion-binding peptides

#### 3.1.1 Biopanning of the phage library

The phage display library was screened using ID elution. After three rounds of biopanning, the phage P/N value increased progressively (Table 1). The P/N values for the three rounds were 50, 130, and 750, respectively, indicating that phage clones capable of binding U(VI) had been enriched.

**Table 1:** Biopanning of phage library.

Rounds of panning	M^U+^ Eluate titer (pfu)	M^−^ Eluate titer (pfu)	P/N
Round 1	5.0 × 10^7^	1.0 × 10^6^	50
Round 2	2.6 × 10^8^	2.0 × 10^6^	130
Round 3	6.0 × 10^8^	8.0 × 10^5^	750

#### 3.1.2 A phage clone exhibits strong affinity toward U(VI)

Twenty plaques randomly selected after the third round of biopanning were tested for affinity toward U(VI). The results showed that all selected phage clones exhibited varying degrees of U(VI) affinity (Table 2). Among them, 11 clones showed relatively high affinity (P/N ≥ 10). Six clones (7-1, 7-5, 7-8, 7-10, 7-12, and 7-16) had P/N values greater than 30. Clone 7-5 exhibited the highest P/N value of 47, indicating the strongest affinity toward U(VI).

**Table 2:** Affinity determination of phage for U(VI).

Phage No.	Amplified liquid titer	M^U+^ Eluate titer (pfu)	M^−^ Eluate titer (pfu)	P/N
7-1	4 × 10^13^	2.8 × 10^7^	7 × 10^5^	40
7-2	2 × 10^13^	9 × 10^6^	6.5 × 10^5^	13.8
7-3	1.2 × 10^13^	3.5 × 10^6^	7 × 10^5^	5
7-4	3 × 10^13^	4.2 × 10^6^	4.8 × 10^5^	9.3
7-5	4 × 10^13^	8.5 × 10^7^	1.8 × 10^6^	47
7-6	1.2 × 10^13^	4.7 × 10^6^	2.8 × 10^7^	1.7
7-7	>10^14^	8.5 × 10^6^	2.3 × 10^6^	3.7
7-8	4.5 × 10^13^	3.4 × 10^7^	1 × 10^6^	34
7-9	3 × 10^13^	1.3 × 10^6^	9.6 × 10^5^	1.4
7-10	3.5 × 10^13^	1.8 × 10^7^	4.4 × 10^5^	41
7-11	1.5 × 10^15^	2.5 × 10^6^	2.6 × 10^5^	10
7-12	0.8 × 10^13^	2.8 × 10^7^	6.1 × 10^5^	46
7-13	1.2 × 10^13^	2.5 × 10^7^	1.1 × 10^6^	25
7-14	1.6 × 10^13^	1.2 × 10^7^	4.6 × 10^5^	26
7-15	5.8 × 10^13^	1.13 × 10^7^	5.6 × 10^6^	2
7-16	3.5 × 10^13^	2.5 × 10^7^	6.5 × 10^5^	39
7-17	2.5 × 10^14^	1.8 × 10^6^	1.4 × 10^6^	1.3
7-18	4.5 × 10^13^	4 × 10^6^	5.2 × 10^5^	7.7
7-19	2.8 × 10^14^	5.7 × 10^6^	1.4 × 10^6^	4.1
7-20	6 × 10^14^	6.6 × 10^6^	5.5 × 10^5^	12

#### 3.1.3 Sequence analysis of phage clones

**Figure 1:**
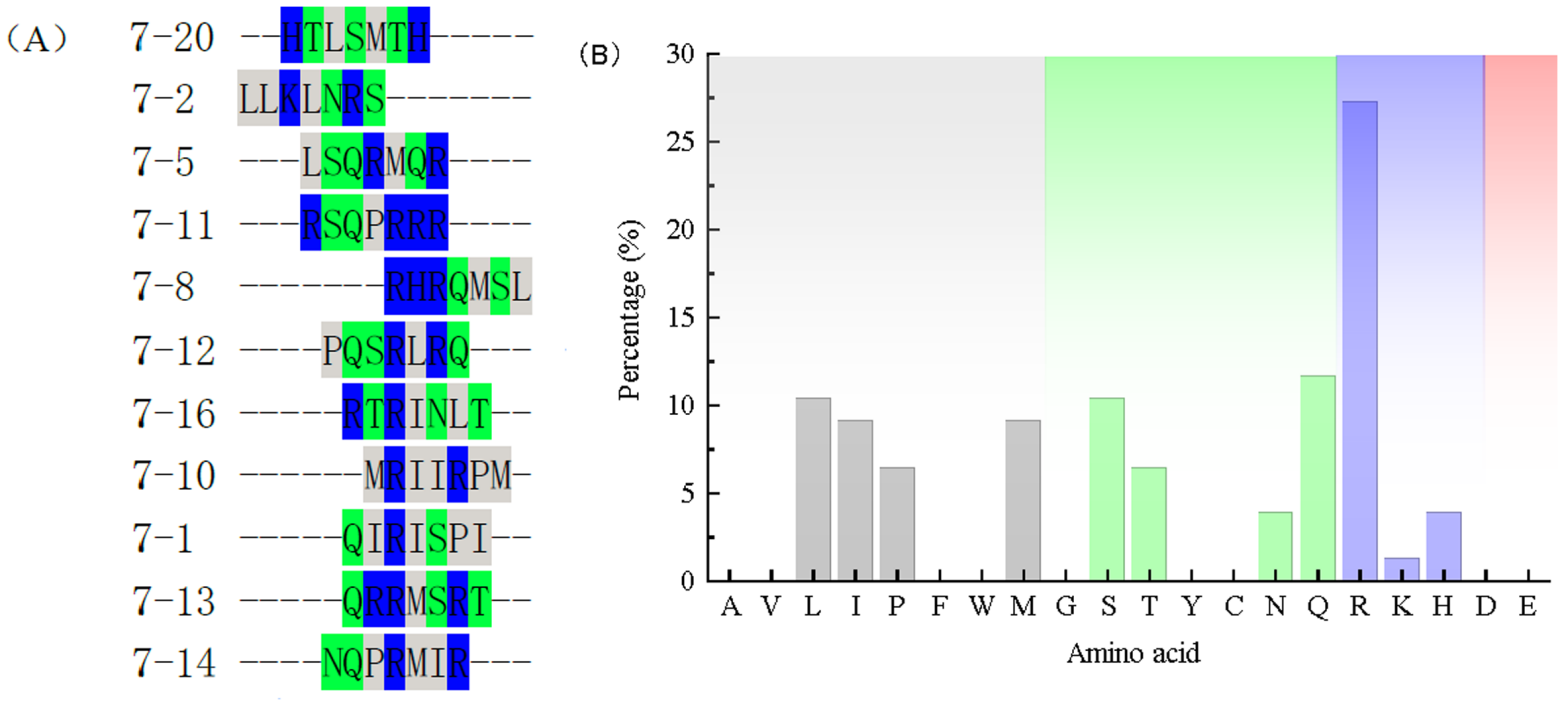
Heptapeptide sequences **(A)** and amino acid composition **(B)** of phage clones after biopanning. Amino acids are color-coded according to the physicochemical properties of their side chains: gray for non-polar (hydrophobic), green for polar uncharged (hydrophilic), blue for positively charged (basic), and red for negatively charged (acidic).

Eleven phage clones were sequenced, and their corresponding heptapeptide sequences were determined (Figure 1A). Sequence analysis revealed that the 11 sequences contained 77 amino acid residues, comprising only 11 distinct amino acids. Regarding amino acid composition (Figure 1B), basic and polar amino acids were highly enriched. Among them, arginine (Arg) showed the highest frequency, accounting for 27.27% of the total residues, followed by glutamine (Gln, 11.69%), leucine (Leu, 10.39%), serine (Ser, 10.39%), isoleucine (Ile, 9.09%), and methionine (Met, 9.09%). No negatively charged acidic amino acids (aspartic acid or glutamic acid) were present.

#### 3.1.4 Peptides exhibit selective binding toward uranyl ions

**Figure 2:**
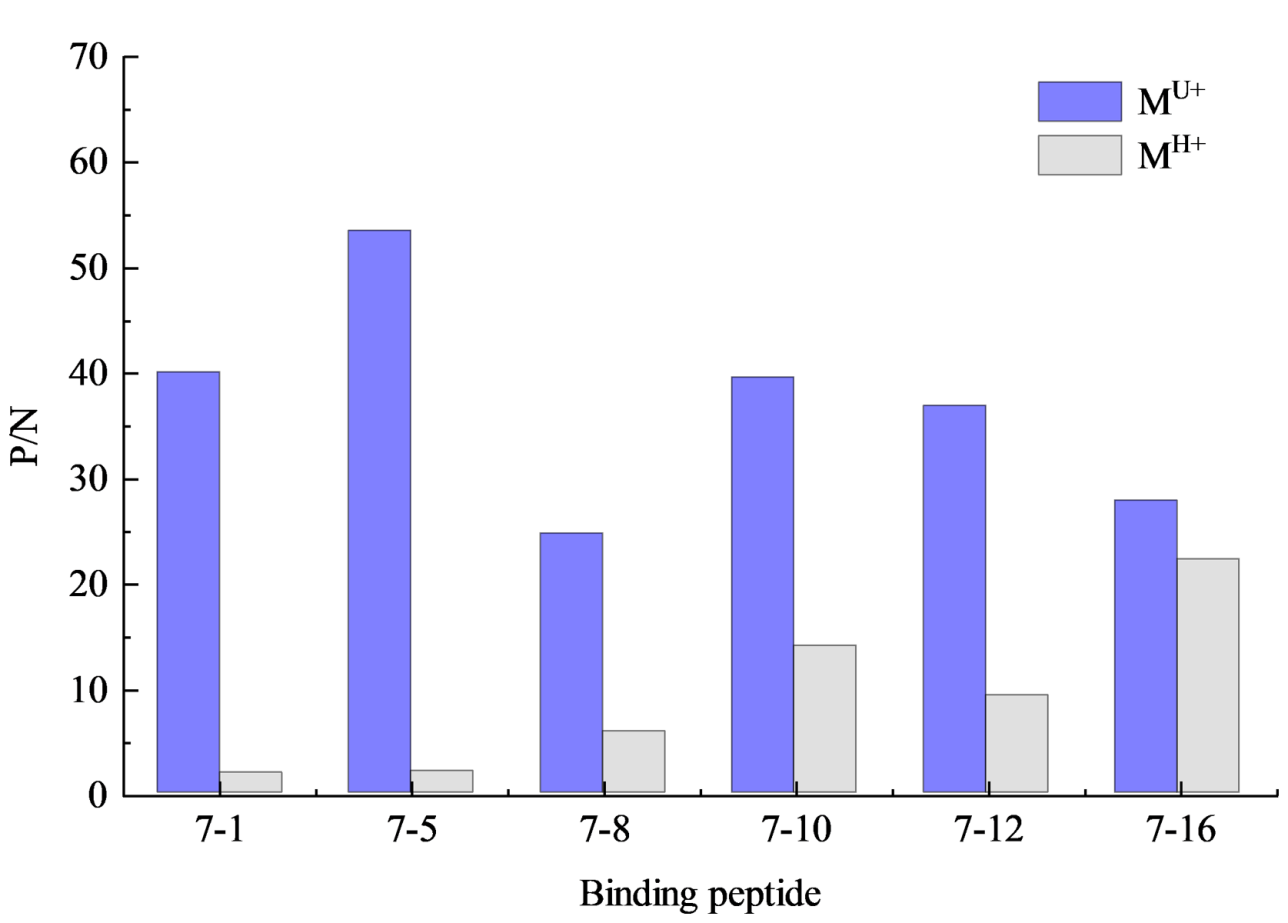
Selectivity of binding peptides toward U(VI) evaluated by P/N ratio.

As multiple heavy metal ions often coexist in nuclear wastewater, we further evaluated the binding selectivity of six representative peptides (7-1, 7-5, 7-8, 7-10, 7-12, and 7-16) toward U(VI) versus other metal ions. In the presence of competing metal ions, all six peptides exhibited certain selectivity for U(VI) (Figure 2). Among them, peptides 7-1 and 7-5 showed the most pronounced selectivity, with P/N values of 40 and 53.5 for U(VI), respectively, compared to 1.86 and 2.07 for the mixed metal ions.

### 3.2 Surface display of U(VI)-binding peptides on yeast cells

#### 3.2.1 Transformation of binding peptide 7-5 into yeast cells

The gene encoding binding peptide 7-5 was cloned into the surface display vector pYD1 to generate the recombinant plasmid pYD-7-5. The plasmid was transformed into *S. cerevisiae* EBY100 cells using 1 μg of recombinant plasmid, 10 μg of ssDNA, and 500 μL of LiAc/PEG solution. Transformants were selected on MD plates. After 48 h, 100 μL of competent cells yielded 3.4 × 10^4^ transformants, corresponding to a transformation efficiency of 3.4 × 10^4^ CFU/μg DNA.

#### 3.2.2 Confirmation of transformants

**Figure 3:**
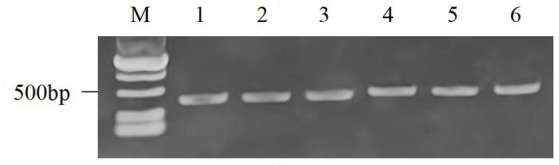
PCR identification of *Saccharomyces cerevisiae* transformant. M: DL5000 Marker; 1–6: PCR products.

Six yeast transformant colonies were randomly selected, and plasmid DNA was extracted for PCR confirmation. All colonies produced an amplicon of approximately 400 bp, consistent with the expected size, as shown by agarose gel electrophoresis (Figure 3). Sequencing results confirmed that the amplicon sequence matched the target fragment, indicating successful introduction of the recombinant plasmid pYD-7-5 into *S. cerevisiae* cells. The resulting strain was designated EBY100-7-5.

**Figure 4:**
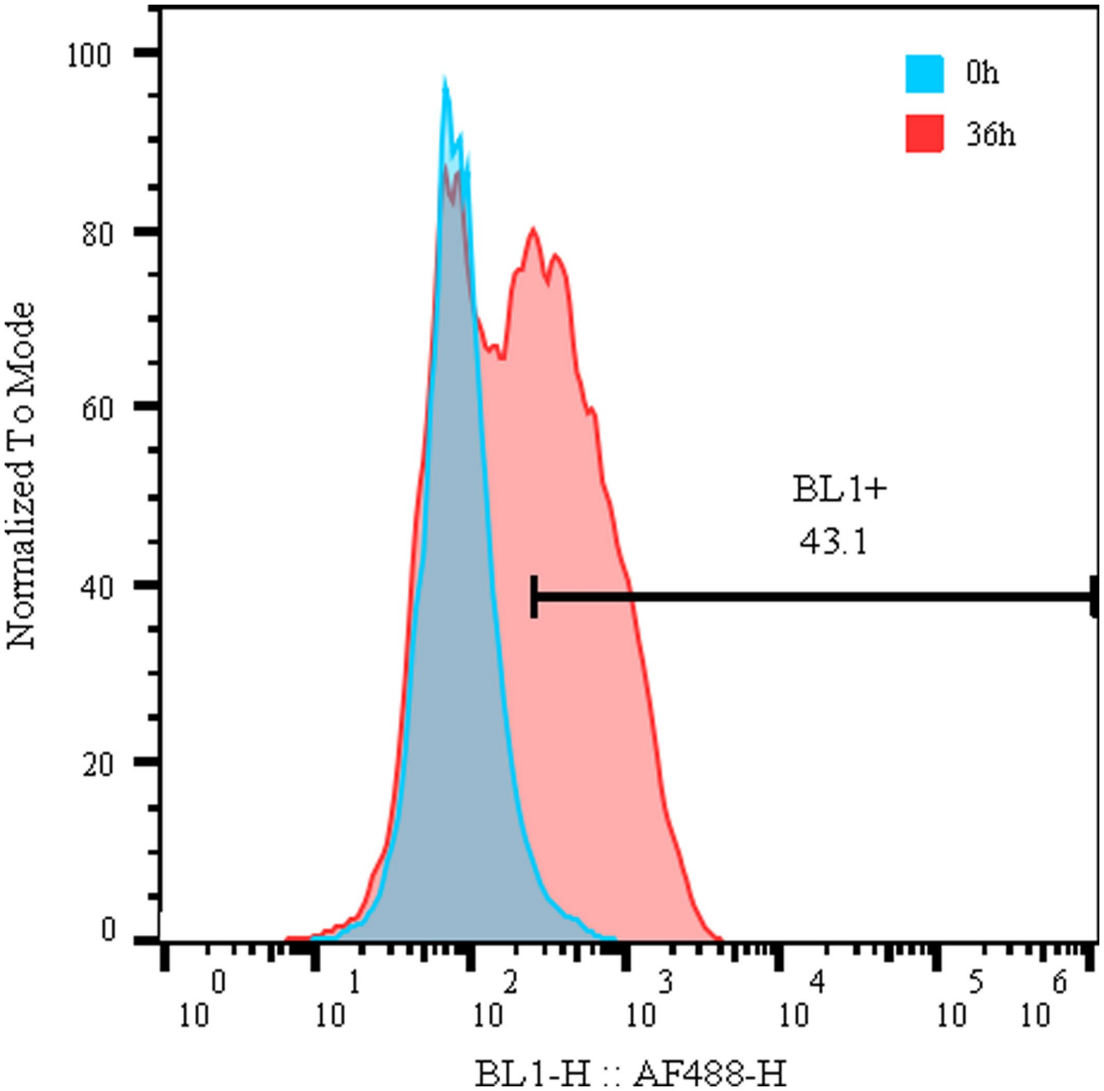
Flow cytometric analysis of cell-surface display of the Aga2p-7-5 fusion protein. Non-induced cells (0 h) served as a negative control. The percentages of positive cells are indicated (non-induced: 3.5%; induced: 43.1%).

Flow cytometric analysis confirmed cell-surface display of the fusion protein (Figure 4). After galactose induction for 36 h, approximately 43.1% of EBY100-7-5 cells exhibited positive fluorescence, whereas non-induced control cells showed only 3.5% positive staining. These results demonstrate that the 7–5 peptide was successfully displayed on the yeast cell surface.

### 3.3 Adsorption performance of the engineered yeast strain toward uranium

#### 3.3.1 Effect of initial solution pH on U(VI) adsorption by yeast

**Figure 5:**
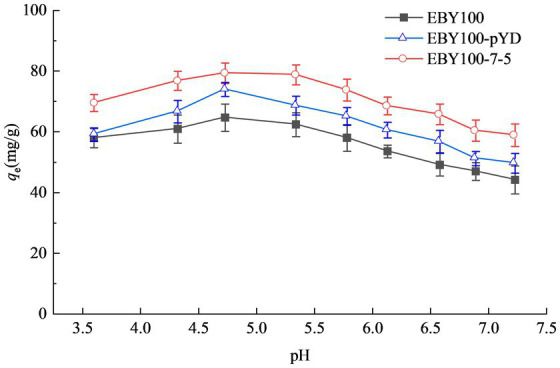
Effect of solution pH on U(VI) adsorption onto yeast cells (*V* = 50 mL; *m* = 0.03 g; *C*_0_ = 50 mg/L; *T* = 298.15 K).

Over the pH range of 3.50–7.23, EBY100-7-5 exhibited significantly higher U(VI) adsorption capacity than both control strains, EBY100 and EBY100-pYD (Figure 5). At pH 4.73, EBY100-7-5 reached its maximum adsorption capacity of 79.33 ± 3.39 mg/g, which was 22.69% higher than that of EBY100 (*p* < 0.05) and also significantly higher than that of EBY100-pYD (73.97 ± 2.30 mg/g, *p* < 0.05). These results confirm that peptide 7-5 provides additional binding sites beyond the His-tag, contributing significantly to U(VI) adsorption at pH 4.73.

#### 3.3.2 Adsorption kinetics of U(VI) by yeast

**Figure 6:**
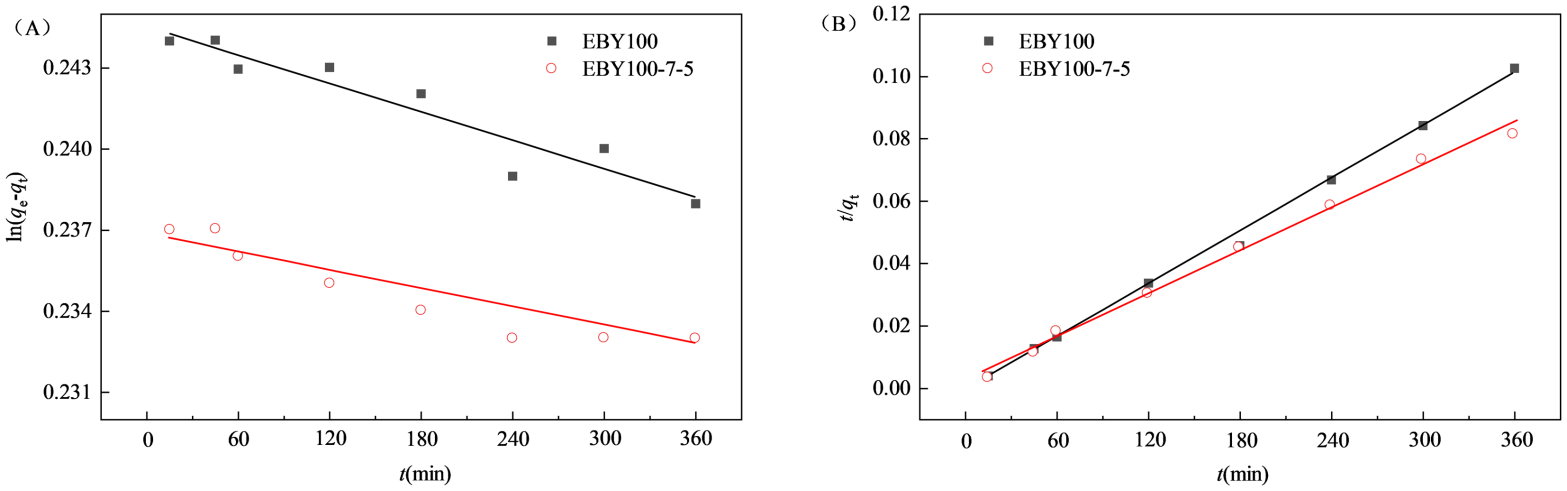
Pseudo-first-order **(A)** and Pseudo-second-order **(B)** adsorption kinetics of yeast cells.

The pseudo-second-order model better described the U(VI) adsorption behavior of both yeast strains (Figure 6). For EBY100 and EBY100-7-5, the correlation coefficients (*R*^2^) were above 0.99, and the calculated equilibrium adsorption capacities (*q_2,cal_*: 70.72 mg/g for EBY100 and 79.81 mg/g for EBY100-7-5) agreed well with the experimental values (*q*_e,exp_: 67.45 ± 2.82 mg/g and 78.36 ± 3.56 mg/g, respectively) (Table 3). The adsorption kinetics follow the pseudo-second-order model (*R*^2^ > 0.99; Wang et al., 2022), suggesting that functional groups on the cell surface may serve as the key adsorption sites.

**Table 3:** Kinetic parameters for adsorption of U(VI) onto yeast cells.

Yeast cells	*q*_e.exp_ (mg/g)	Pseudo-first-order model			Pseudo-second-order model		
		*q*_1,cal_ (mg/g)	*k*_1_ (/min)	*R* ^2^	*q*_2,cal_ (mg/g)	*k*_2_ g/(mg·min)	*R* ^2^
EBY100	67.45 ± 2.82^a^	58.81	7.6 × 10^−2^	0.767	70.72	2.9 × 10^−1^	0.993
EBY100-7-5	78.36 ± 3.56^b^	72.31	3.7 × 10^−2^	0.714	79.81	4.2 × 10^−1^	0.991

*q*_e.exp_ values are presented as mean ± SD (*n* = 3). Different superscript letters indicate significant difference (*p* < 0.05).

Regarding the adsorption rate, EBY100-7-5 exhibited a higher rate constant (0.42 g/(mg·min)) than EBY100 (0.29 g/(mg·min)). Further demonstrating that the surface-displayed binding peptides effectively enhance U(VI) adsorption.

#### 3.3.3 Yeast adsorption isotherms for U(VI)

**Figure 7:**
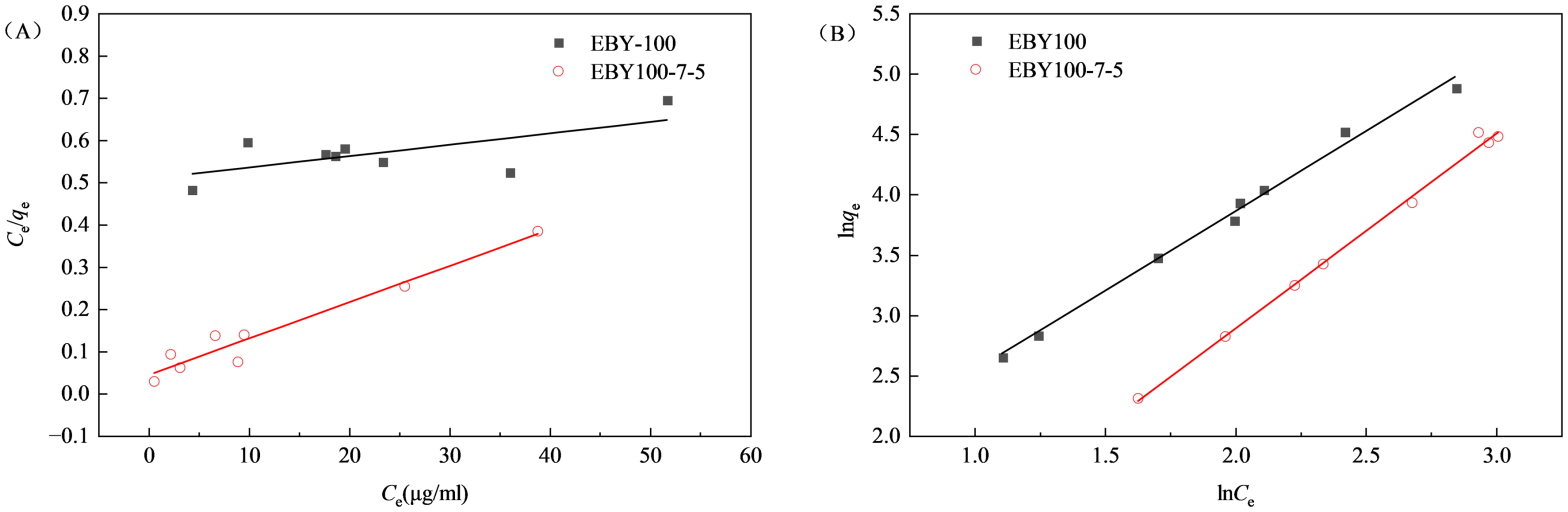
Langmuir **(A)** and Freundlich **(B)** adsorption isotherms of U(VI) on yeast cells.

U(VI) adsorption by both yeast strains followed the Freundlich isotherm model (*R*^2^ > 0.99), indicating heterogeneous surface adsorption rather than ideal monolayer coverage (Figure 7). The Freundlich capacity constant *K*_F_ for EBY100-7-5 was 30.98, which is approximately 45.58% higher than that of EBY100 (21.28) (Table 4). Thus, the surface-displayed binding peptides provided abundant U(VI) binding sites, enhancing the adsorption capacity of the engineered strain.

**Table 4:** Parameters of Langmuir and Freundlich isotherm for adsorption of U(VI) onto yeast cells.

Yeast cells	Langmuir isotherm			Freundlich isotherm		
	*K*_L_	*q*_m_/(mg/g)	*R* ^2^	*K*_F_	*n*	*R* ^2^
EBY100	0.032	217.54	0.84	21.28	1.08	0.991
EBY100-7-5	0.039	114.54	0.69	30.98	2.94	0.995

#### 3.3.4 Thermodynamics of yeast U(VI) adsorption

Thermodynamic parameters for U(VI) adsorption are listed in Table 5. Positive ΔH and ΔS values indicate an endothermic, entropy-increasing process. Negative ΔG values, which became more negative with rising temperature, indicate spontaneous adsorption favored by higher temperatures (Girish, 2025). EBY100-7-5 exhibited larger absolute ΔG values than EBY100, indicating stronger thermodynamic spontaneity. These findings, together with the higher *k*_2_ value for EBY100-7-5, demonstrate that the surface-displayed binding peptide enhanced U(VI) adsorption.

**Table 5:** Adsorption thermodynamic parameters of U(VI) adsorption onto yeast cells.

Yeast cells	Δ*H* (kJ·mol^−1^)	Δ*S* (J·mol^−1^·K^−1^)	Δ*G* (kJ·mol^−1^)			
			288.15(K)	298.15(K)	308.15K	313.15K
EBY100	4.99	92.10	−21.54	−22.46	−23.39	−23.84
EBY100-7-5	3.49	88.06	−21.62	−22.77	−23.65	−24.09

### 3.4 Selective adsorption performance of the engineered yeast strain toward U(VI)

**Figure 8:**
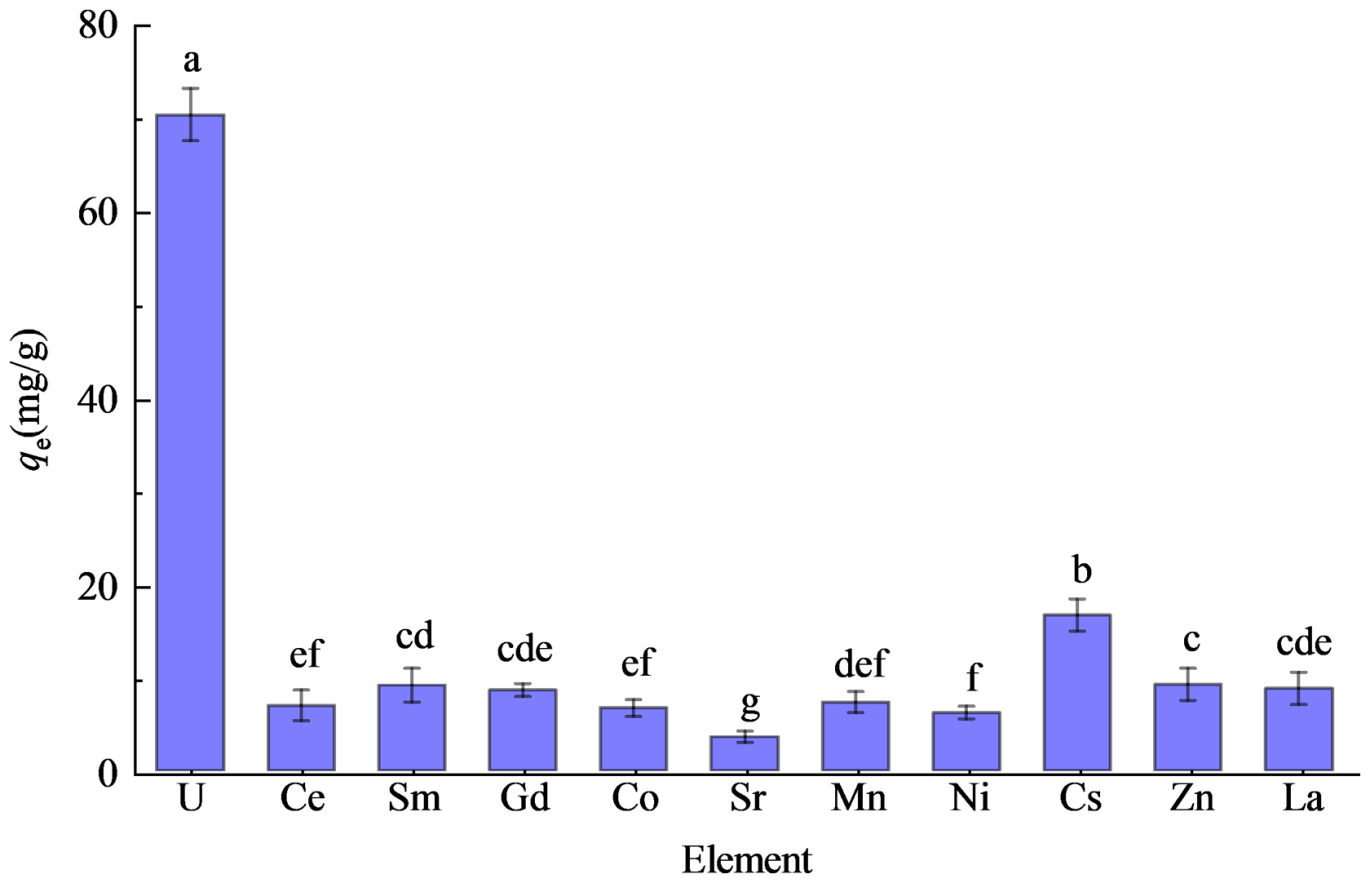
Selective adsorption of U(VI) by EBY100-7-5 cells (*V* = 50 mL; *m* = 0.03 g; *C*_0_ = 50 mg/L; *T* = 298.15 K). Data are mean ± SD (*n* = 3). Different letters indicate significant differences (*p* < 0.05).

EBY100-7-5 showed significantly higher adsorption capacity for U(VI) (approximately 70.61 ± 2.85 mg/g) than for other metal ions (*p* < 0.05) (Figure 8). Cs(I) adsorption was approximately 16.97 ± 1.72 mg/g, while all other ions were below 10 mg/g. These results demonstrate that EBY100-7-5 exhibits good selective adsorption for U(VI) in the presence of competing metal ions.

### 3.5 Scanning electron microscopy and energy dispersive spectroscopy analysis of U(VI) adsorption by the engineered yeast strain

**Figure 9:**
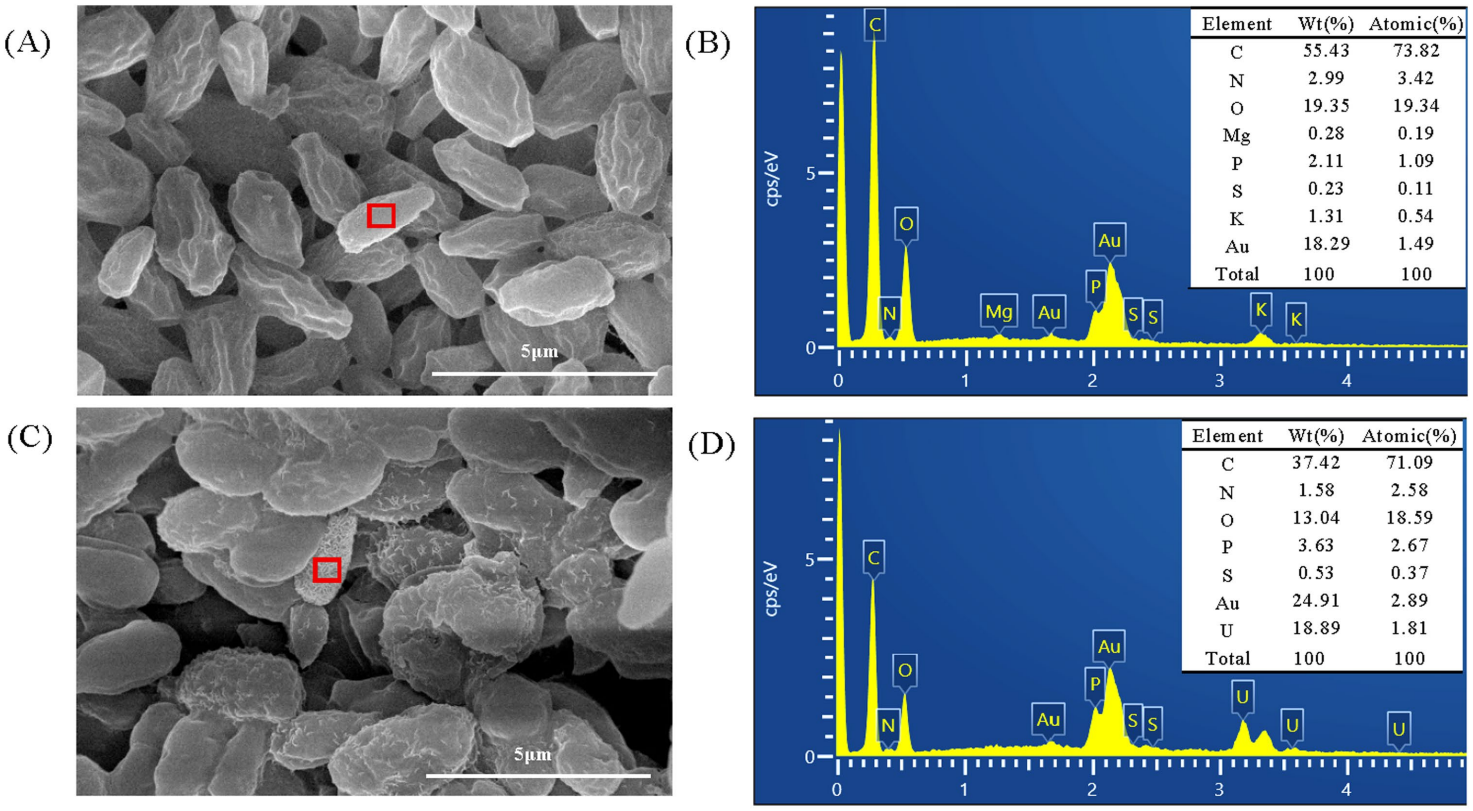
SEM-EDS analysis of engineered yeast cells before **(A,B)** and after **(C,D)** U(VI) adsorption.

Before U(VI) adsorption, yeast cells exhibited a smooth and clean surface (Figure 9). After adsorption, abundant particulate deposits and U(VI) signals were detected on the cell surface by SEM-EDS, supporting the accumulation of U-containing species on the engineered yeast cell surface after adsorption.

## 4 Discussion

### 4.1 Compositional characteristics of uranyl ion-binding peptides

Sequence analysis of the selected phage clones revealed a clear selective preference in amino acid usage, characterized by a marked enrichment of arginine. Acidic residues (aspartic acid and glutamic acid) were not observed in any of the sequenced peptides under the current screening conditions.

This compositional pattern may be mechanistically relevant. The absence of acidic residues suggests that nonspecific electrostatic adsorption is unlikely to be the primary binding mechanism, although this requires further validation. The marked enrichment of arginine, together with the presence of serine, is consistent with a coordination-based recognition mode, although direct evidence is lacking. The guanidino group of arginine and the hydroxyl group of serine could potentially serve as ligands for U(VI) complexation, but this remains to be verified. These findings provide a preliminary basis for understanding the selective recognition of uranyl ions by the isolated peptides.

### 4.2 Selectivity of the binding peptide toward uranyl ions

pH 4.73 was selected as the optimal condition for subsequent experiments because it yielded the highest U(VI) adsorption capacity for EBY100-7-5. At this pH, uranyl ions do not exist solely as free UO_2_^2+^ in solution, but rather coexist mainly as free UO_2_^2+^ and their oligomeric hydroxyl complexes (Langmuir, 1997). Considering the amino acid composition bias from sequence analysis, it is possible that the selectivity for U(VI) is related to a specific coordination environment formed by the peptide side chains. The ionic radius, charge density, and coordination geometry of U(VI), particularly the structural features of its hydroxyl complexes formed under weakly acidic conditions, may be more compatible with this environment than those of competing metal ions, potentially enabling specific binding. It should be noted, however, that under acidic conditions, arginine side chains are generally protonated, and their actual role in coordination remains unclear. Collectively, these observations do not contradict the possibility that selective binding depends on specific spatial coordination rather than simple electrostatic adsorption, but further evidence is required. The exact binding mechanism requires further validation through spectroscopic analyses, mutagenesis studies, or computational modeling.

Regarding the selective adsorption experiment (Figure 8), it should be noted that the reported adsorption capacities of EBY100-7-5 represent its total performance as a whole-cell biosorbent, without background subtraction, as this study aims to evaluate its practical application potential. Furthermore, data from the wild-type EBY100 control under the same competitive conditions were not included in Figure 8, since wild-type yeast lacks specific metal-binding selectivity and exhibits only non-specific electrostatic interactions. Nevertheless, the absence of this control is acknowledged as a limitation.

After cell-surface display of the U(VI)-binding peptide 7–5 on *Saccharomyces cerevisiae*, the recombinant strain EBY100-7-5 exhibited significantly higher adsorption capacity for U(VI) than for other competing metal ions (*p* < 0.05), further confirming the peptide’s selectivity toward uranyl ions. Unlike conventional chemical adsorbents such as amidoxime-functionalized (Bai et al., 2016; Shu et al., 2024; Ahmad et al., 2021), phosphonic acid-functionalized (Tolba et al., 2026), and chitosan-based materials (Li et al., 2022; Zhou et al., 2026; Huang et al., 2026), which rely on functional group coordination and suffer from interference by coexisting ions like vanadium, calcium, and carbonate (Shu et al., 2024; Ahmad et al., 2021). In contrast, the present system achieves high selectivity through specific molecular recognition between the peptide’s three-dimensional structure and the uranyl ion. Moreover, the recombinant yeast cells are simple and inexpensive to prepare, enable mild recovery by centrifugation, and offer a biotechnological route distinct from traditional chemical materials for selective uranyl separation.

### 4.3 Application of cell surface display technology for the adsorption of heavy metal ions

Intracellular expression of metal-binding peptides suffers from poor metal ion accessibility and dependence on cell viability. Cell surface display overcomes these limitations by anchoring exogenous peptides on the host cell surface, allowing direct interaction with metal ions and avoiding transmembrane transport and associated mass transfer resistance. This increases adsorption rates and facilitates metal recovery without cell disruption (Zhang et al., 2022).

Additionally, surface-displayed binding peptides confer enhanced adsorption capacity for metal ions. For instance, palladium-binding peptides achieved 125 mg/g for Pd(II) (Mashangoane and Chirwa, 2022), and cadmium-binding peptides increased Cd^2+^ adsorption by 35% (Wang et al., 2024). In the present study, high-copy-number display of the uranyl-binding peptide increased effective binding sites, enhancing U(VI) adsorption capacity. Importantly, the selectivity of the original peptide was retained after surface display, endowing the engineered strain with excellent selective adsorption performance in the presence of competing metal ions. Thus, cell surface display may serve as a promising strategy for developing selective biosorbents applicable to uranyl ions.

Beyond technical performance, the potential environmental risks associated with the application of genetically modified microorganisms must also be considered. Currently, although the application of genetically modified yeast in environmental remediation is increasing, most studies remain at the laboratory scale. Despite *S. cerevisiae* being recognized as a Generally Recognized as Safe (GRAS) microorganism by the U. S. Food and Drug Administration (FDA), the application of engineered strains still requires rigorous biosafety risk assessment and control measures. Specific strategies include: (1) confinement to closed systems to prevent leakage of viable cells into open environments; (2) immobilization using materials such as sodium alginate or polyvinyl alcohol to enhance cell stability and prevent viable cell dispersal; (3) centralized disposal of saturated biomass according to radioactive waste regulations to avoid secondary contamination. These control strategies can effectively mitigate potential ecological risks while leveraging the excellent adsorption performance of the engineered strain.

### 4.4 Enhancement effect and mechanism of adsorption performance

The engineered strain EBY100-7-5 exhibited a 22.69% higher U(VI) adsorption capacity than the control at optimal pH 4.73. Kinetic data showed a good fit to the pseudo-second-order model (*R*^2^ > 0.99), and the higher rate constant of EBY100-7-5 suggests that surface-displayed binding peptides provide additional sites that accelerate U(VI) uptake.

Both strains followed the Freundlich isotherm (*R*^2^ > 0.99), whereas the Langmuir model yielded lower *R*^2^ values, indicating that the adsorption occurs on heterogeneous surfaces (He et al., 2024). This is consistent with the inherent chemical complexity of the yeast cell wall, which contains diverse functional groups. The substantially higher *K*_F_ value of EBY100-7-5 indicates greater adsorption capacity, while its higher absolute Δ*G* value reflects enhanced thermodynamic spontaneity. Together with the kinetic results, these findings support that surface-displayed binding peptides increase effective binding sites and improve adsorption performance for U(VI).

## 5 Conclusion

In this study, a uranyl-binding peptide (7-5) with high affinity and selectivity for U(VI) was isolated from a phage-displayed heptapeptide library. The peptide was displayed on the surface of *S. cerevisiae* EBY100, generating the engineered strain EBY100-7-5.

Adsorption studies showed that EBY100-7-5 achieved a maximum U(VI) adsorption capacity 22.69% higher than that of the control strain EBY100. The adsorption behavior followed the pseudo-second-order kinetic model (*R*^2^ > 0.99) and the Freundlich isotherm model (*R*^2^ > 0.99). Both the rate constant and the adsorption capacity constant (*K*_F_) of EBY100-7-5 were higher than those of the control, confirming that the surface-displayed binding peptides provide efficient binding sites and enhance adsorption kinetics and capacity.

Thermodynamic analysis indicated that the adsorption process is spontaneous, endothermic, and entropy-driven, with EBY100-7-5 exhibiting stronger thermodynamic spontaneity than the control. In the presence of 11 competing metal ions, EBY100-7-5 showed significantly higher adsorption capacity for U(VI) than for other ions, demonstrating effective selective adsorption. These findings provide a novel yeast-based biosorbent for the treatment of uranium-containing radioactive wastewater.

## Data Availability

The raw data supporting the conclusions of this article will be made available by the authors, without undue reservation.
