# Supplementary figures and images for: A novel uranyl ion-binding peptide enhances U(VI) adsorption capacity and selectivity of Saccharomyces cerevisiae
Source: Front Microbiol. 2026 Jul 13;17:1869418. doi: 10.3389/fmicb.2026.1869418 (PMC13402460; doi:10.3389/fmicb.2026.1869418)

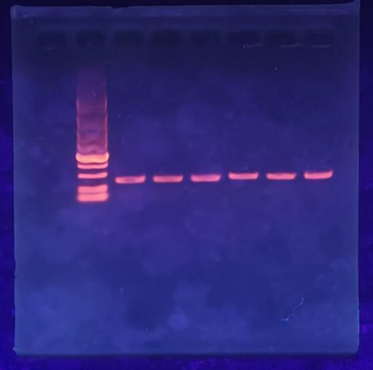

Supplement: Supplementary file 1 [file Image_1.TIF]

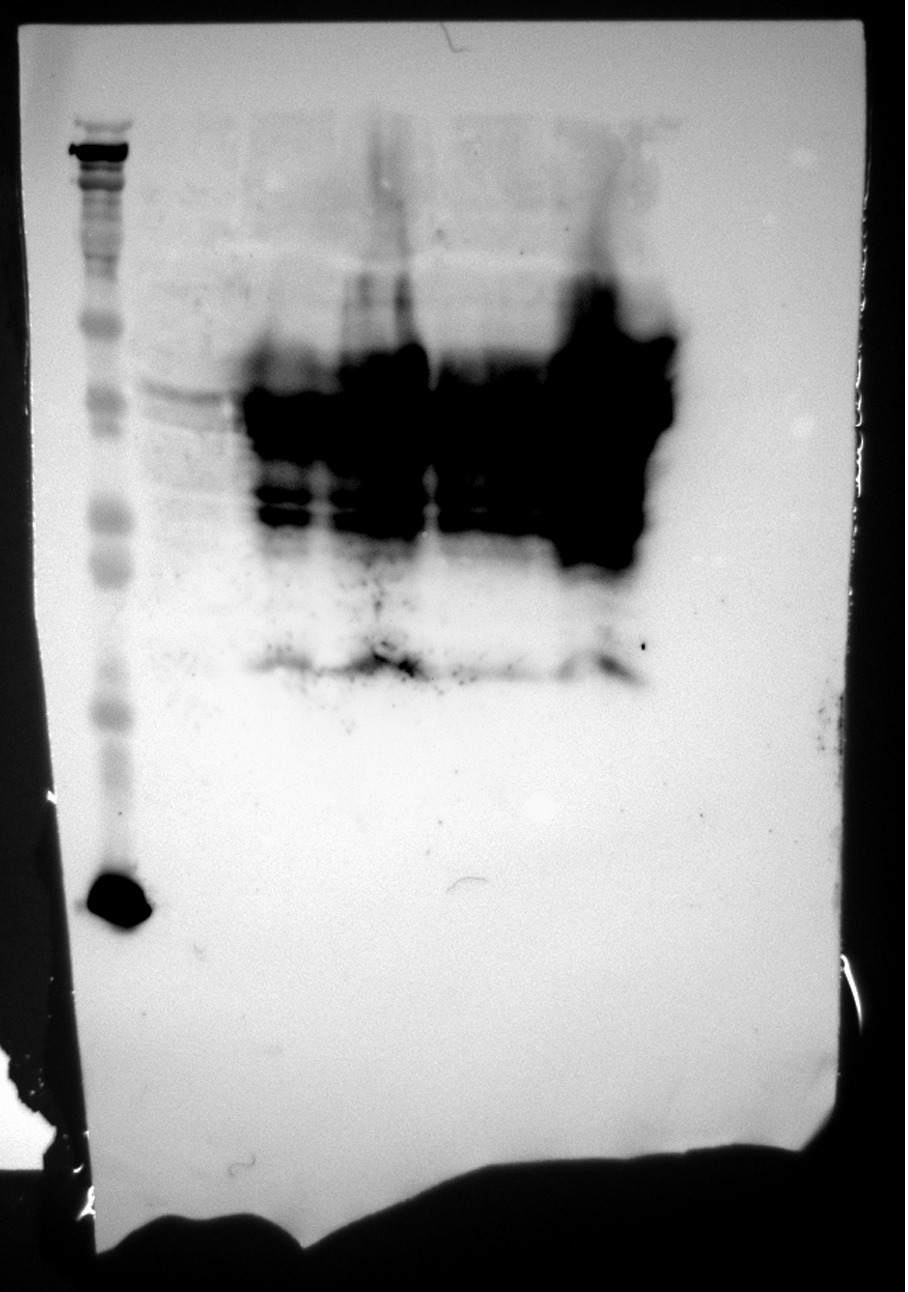

Supplement: Supplementary file 2 [file Image_2.TIF]
